# Decreased Apoptotic Rate of Alveolar Macrophages of Patients with Idiopathic Pulmonary Fibrosis

**DOI:** 10.1155/2012/981730

**Published:** 2012-06-25

**Authors:** Fotios Drakopanagiotakis, Areti Xifteri, Evaggelos Tsiambas, Andreas Karameris, Konstantina Tsakanika, Napoleon Karagiannidis, Demetrios Mermigkis, Vlasis Polychronopoulos, Demosthenes Bouros

**Affiliations:** ^1^3rd Respiratory Medicine Department, Sismanoglio General Hospital, 15126 Marousi, Greece; ^2^Department of Pathology and Computerized Image Analysis, 417 NIMTS Hospital, 11521 Athens, Greece; ^3^Bronchoalveolar Lavage Unit, Sismanoglio General Hospital, 15126 Marousi, Greece; ^4^Department of Pneumonology, University Hospital of Alexandroupolis and Medical School of Democritus University of Thrace, 68100 Alexandroupolis, Greece

## Abstract

*Introduction*. Increased apoptosis of epithelial cells and decreased apoptosis of myofibroblasts are involved in the pathogenesis of IPF. The apoptotic profile of alveolar macrophages (AMs) in IPF is unclear. *Aim*. To investigate whether AMs of patients with IPF exhibit a different apoptotic profile compared to normal subjects. *Methods*. We analyzed, by immunohistochemistry, the expression of the apoptotic markers fas, fas ligand , bcl-2, and bax in AM obtained from bronchoalveolar lavage fluid (BALF) of 20 newly diagnosed, treatment-naive IPF patients and of 16 controls. Apoptosis of AM was evaluated by Apoptag immunohistochemistry. IPF patients received either interferon-g and corticosteroids or azathioprine and corticosteroids for six months. *Results*. BALF AMs undergoing apoptosis were significantly less in IPF patients. No difference was found in the expression of fas or fas ligand, bcl-2 and bax between IPF and control group. No difference was found between the respiratory function parameters of the two treatment groups after six months. A positive correlation was found between the number of bcl-2 positive stained macrophages and DLCO after treatment. *Conclusions*. The decreased apoptotic rate of AM of patients with IPF is not associated with decreased expression of apoptosis mediators involved in the external or internal apoptotic pathway.

## 1. Introduction

Idiopathic pulmonary fibrosis (IPF) is a chronic diffuse lung disease, characterised by progressive deterioration which ultimately leads to death [1]. 

Apoptosis is an important physiological process for the development and the maintenance of tissue homeostasis which ensures a balance between cellular proliferation and turnover in nearly all tissues [2]. Apoptosis can be activated by two pathways.

- The extrinsic or death-receptor pathway which involves the activation of death receptors present in the cell membrane, which are activated by death ligands. Fas and TNF-receptor 1 are the two most known death receptors. Connection of death Ligand, such as Fas Ligand to its death receptor leads to receptor polymerisation and activation of adaptor proteins called “activated death domains” which activate procaspase molecules to caspases.
- The intrinsic pathway which is associated to an increase of mitochondrial permeability and is activated by cellular “stress.” Cellular stress leads to reduced expression of antiapoptotic mitochondrial proteins (bcl-2, bcl-x) and to increased expression of proapoptotic mitochondrial proteins (bak, bax, bim). The reduction of anti-apoptotic proteins causes an increase in mitochondrial membrane permeability and subsequent outflux of cytochrome c to the cytoplasm.

Increased apoptosis of epithelial cells resulting to inefficient reepithelialization [3] and resistance to apoptosis of fibroblasts and myofibroblasts associated with increased fibrosis has been described in lung biopsies of patients with IPF [2]. 

Macrophages play an important role for the removal of apoptotic cells, a process called “efferocytosis” [4]. Apoptotic macrophages have been reported to induce the apoptosis of normal macrophages and exaggerate lung fibrosis in experimental models and participate in the pathogenesis of fibrotic lung diseases such as the Hermansky-Pudlak syndrome [5]. However, the apoptotic profile of macrophages in IPF is not clear.

Bronchoalveolar lavage is a useful tool for research purposes in patients with idiopathic pulmonary fibrosis. Different cell populations can be obtained from bronchoalveolar lavage, in order to be evaluated in relation to the cytokines and growth factors, which they produce, as well as to their apoptotic behavior [6]. 

In the current study we investigated the expression of apoptotic markers in naive alveolar macrophages obtained from BAL of IPF patients and of normal subjects. Furthermore, we tried to correlate apoptotic markers' expression with clinical parameters.

## 2. Materials and Methods

### 2.1. Subjects

The study group consisted of twenty patients with newly diagnosed IPF who were admitted to “Sismanoglio” hospital from 2003 until 2007. The patients had not received any prior treatment for IPF. The diagnosis of IPF was established either by a surgical biopsy showing a usual interstitial pneumonia pattern or using the criteria of the American Thoracic Society [7–9]. 

Patients with acute exacerbation of IPF, infection, or uncontrolled heart disease were not included in the study. The control group consisted of otherwise healthy individuals who were submitted to bronchoscopy for various reasons, mainly chronic cough and hemoptysis. No subject in the control group suffered from malignant, inflammatory, or interstitial lung disease.

### 2.2. Study Design

The twenty patients were randomised to receive a combination of interferon-g (IFN*γ*-1b) 200 *μ*g subcutaneously 3 times per week and 10 mg of oral prednisolone daily or 150 mg of oral azathioprine (AZA) and 10 mg of oral prednisolone daily. The study was approved by the Bioethics Committee of Sismanoglio Hospital and all participants gave informed consent.

Patients were evaluated at the beginning, three months and six months after treatment. Lung function testing, HRCT, bronchoscopy, and bronchoalveolar lavage were performed prior to treatment. Lung function testing was performed six months after treatment.

The two patient groups were compared regarding lung function parameters, and their correlation to bronchoalveolar lavage apoptosis markers in AM prior to treatment.

### 2.3. Methods

Fiberoptic bronchoscopy with bronchoalveolar lavage was performed in the newly diagnosed, treatment-naive IPF patients and in the control group according to recommended guidelines and previous reports [10, 11]. Cells were separated from BAL by low-speed centrifugation at 300 g for five minutes at 4°C and were washed three times with cold minimal essential medium (MEM) containing 25 Mm Hepes buffer. Total cell counts were determined using an improved Neubauer counting chamber. Slide preparations for differential percentage counting of the cells were made with a Shandon cytocentrifuge (Cytospin II, Shandon Ltd, Runcorn, Cheshire, UK). The differential count was determined on a stained preparation stained by May-Grunwald Giemsa staining and Papanicolaou staining after counting more than 1000 cells. 

### 2.4. Antibodies and Immunohistochemistry (IHC)

We selected and applied monoclonal antibodies including anti-bcl2 (Dako-Cytomation, Danemark), anti-bax (Dako-Cytomation, Denmark), anti-Fas (CD95) (Novo-Castra, UK) and also anti-fas ligand (Novo-Castra, UK) according to the manufacturer instructions. For detection of apoptosis, we used the Apopt-Ag plus peroxidase in situ apoptosis detection kit (Chemicon International) [12]. 

### 2.5. Evaluation of IHC Results by Computerized Image Analysis (CIA)

**Figure 1:**
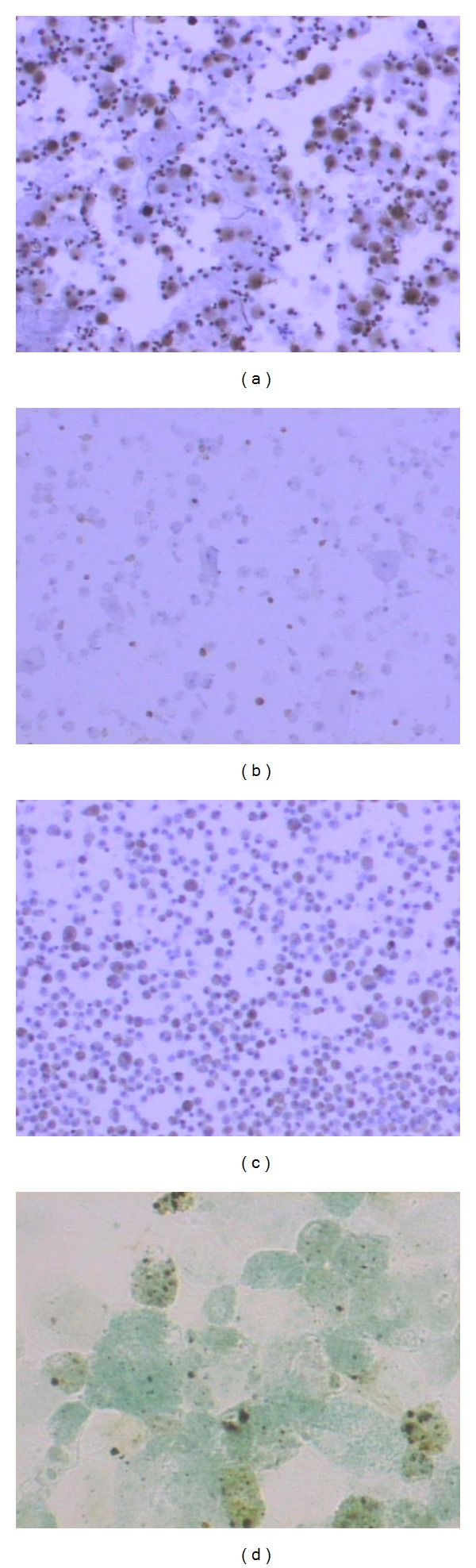
Computerized image analysis of stained BALF macrophages in patients with IPF. Brown colored dots represent the expression of apoptotic markers (objects) in BALF macrophages: (a) bax, (b) bcl-2, (c) fas, (d) ApoptAg.

We performed CIA by using a semiutomated system with the following hardware features: Intel Pentium IV, MATROX II CARD FRAME GRABBER, CAMERA MICROWAVE SYSTEMS (resolution of 800 × 600), microscope Olympus BX-50 and the following software: Windows XP/Image Pro Plus version 3.0-Media Cybernetics 1997. Measurements regarding protein expression of the markers described above were performed in 5 optical fields per case and at magnification of 400 (40 × 10). The brightness values represent levels within a 256-level scale (0–255). In all examined cases, areas of significant cellularity including isolated macrophages or small clusters of them were considered to be eligible for measurements. Semiautomated segmentation was performed by splitting those small clusters. Furthermore, for the evaluation of ApopTag method, the total amount of signals per case was measured (area covering brown staining pattern) at the same magnification (Figure 1).

### 2.6. Statistical Analysis

Analysis of variance was used to assess differences of the *apopt ag signals, apopt ag area and bcl2, bax, fas and fasl density* between patients and controls. Prior to the analysis, the density, signals, and area values were transposed into natural logarithms in order to reduce the within-patient variability. Summary statistics are expressed as in means and 95% confidence intervals. Tests were 2-sided and level of statistical significance was set at 5%. *P* < 0.05 values were considered as significant. For statistical analysis, SPSS version 17 was used.

## 3. Results

Baseline characteristics of the patients are shown in Table 1. Mean age was 69,05 years with a mean duration of symptoms of 14,15 months. Respiratory function was moderately impaired with a mean FVC 71,1% of predicted, diffusion capacity for carbon monoxide 55,2% of predicted and mean PaO_2_ of 72,09 mm Hg.

Neutrophils, eosinophils, and mast cells were significantly increased in the BALF of patients with IPF compared to the control group. BALF of patients with IPF was also characterized by reduced percentage of macrophages (Table 2). There was no difference in bronchoalveolar lavage cell count between the two patient groups at baseline.

**Figure 2:**
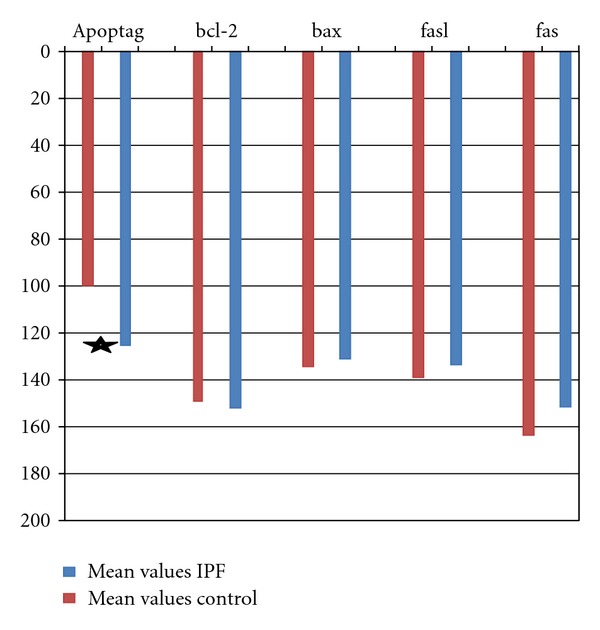
Mean staining densities for apoptotic markers between IPF patients (blue) and control (red). Values range between 0–255 A statistically significant increase in Apoptag density (reduced apoptosis expression) is observed in IPF patients.

**Figure 3:**
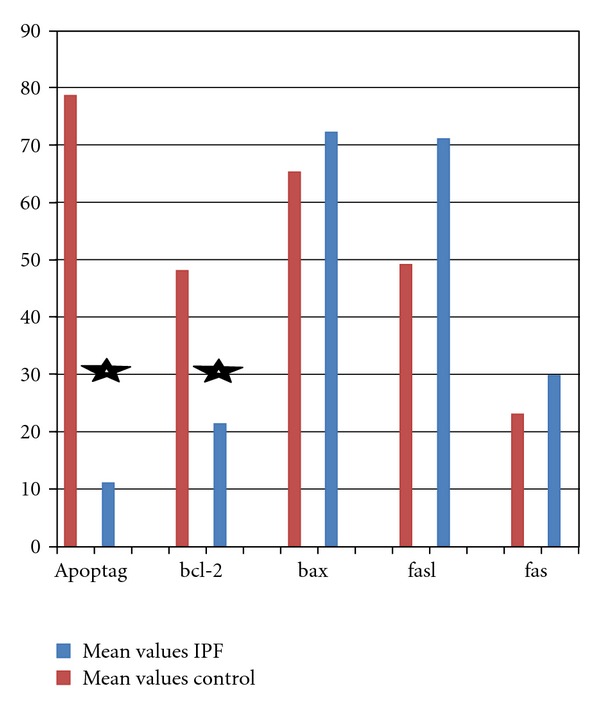
Mean number of positive objects (positive-stained cells) for apoptotic markers between IPF patients (blue) and control (red). A statistically significant increased number of Apoptag objects and of the antiapoptosis marker bcl-2 are observed in the control group compared to IPF patients.

**Figure 4:**
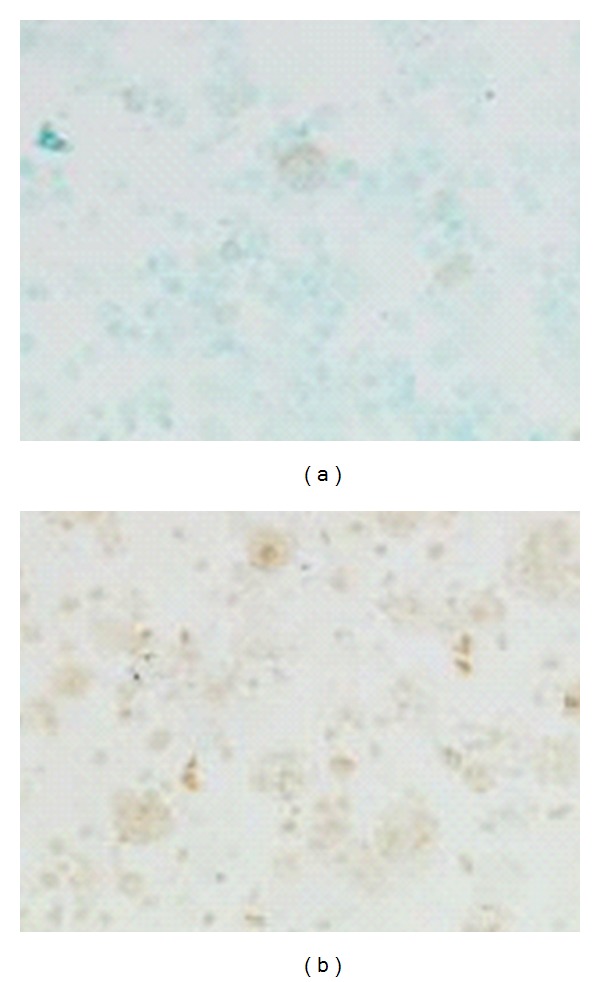
Decreased expression of apoptosis (Apoptag) in BALF macrophages of patients with IPF (a) compared to the control group (b).

We examined the expression of specific apoptotic markers in BALF macrophages of treatment-naive patients and control group, representing activation of the extrinsic (fas, fas ligand) and the intrinsic pathway (bcl-2, bax) and total expression of apoptosis, based on expression of Apoptag; a statistically significant difference was found between the IPF group and control group at presentation regarding expression of Apoptag. Macrophages of patients with IPF showed reduced expression of Apoptag and reduced Apoptag stained area compared to macrophages of the control group (Table 3, Figures 2, 3, and 4).

**Figure 5:**
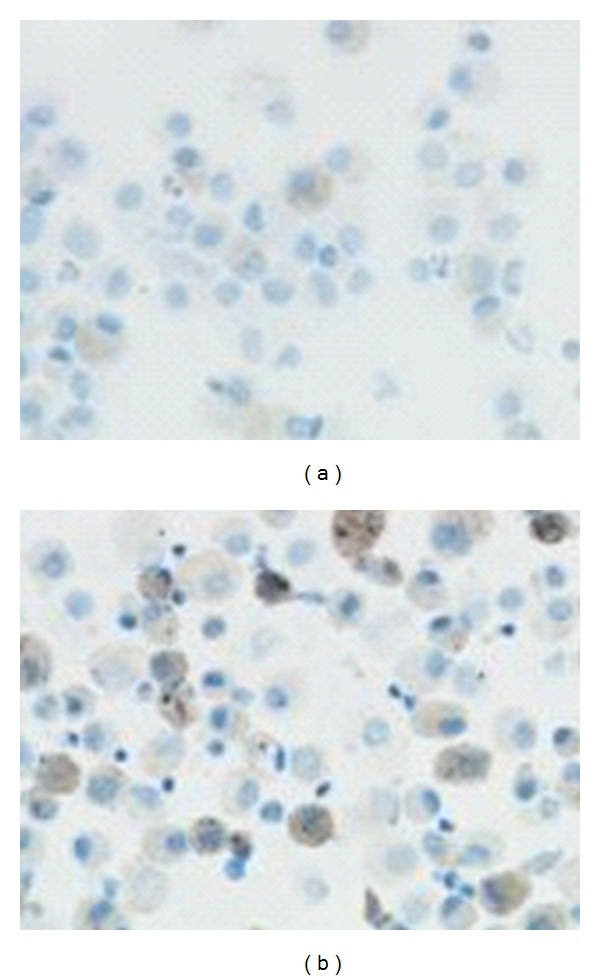
Decreased expression of the antiapoptotic bcl-2 protein in BALF macrophages of patients with IPF (a) compared to the control group (b).

Immunohistochemical staining showed no difference regarding the staining intensity of specific apoptotic markers of either the intrinsic (bcl-2, bax) or extrinsic apoptosis pathway (fas, fasl). However, the number of macrophages of IPF patients expressing the anti-apoptotic protein bcl-2 was significantly less compared to controls (Table 3, Figures 2, 3, and 5).

There were no differences between the interferon-g plus prednisone and azathioprine plus prednisone groups of patients regarding baseline demographic characteristics, smoking habit, clinical presentation, and respiratory function parameters.

Comparison of respiratory function parameters six months after treatment showed no difference between the two patient groups (Table 4).

We tried to correlate the expression of apoptotic markers in macrophages prior to treatment with respiratory function parameters (FVC, FEV1, DLCO) in patients with IPF before and after treatment. Expression of apoptotic markers in BALF did not correlate to pulmonary function parameters neither before nor after treatment with the exception of a positive relation between the number of bcl-2 positive stained macrophages and DLCO after treatment (*r* : 0.646, *P* : 0.032) (Table 5).

## 4. Discussion

In our study we demonstrated decreased apoptosis of bronchoalveolar lavage macrophages in patients with IPF compared to controls. This difference could not be attributed to the increased activation of the external apoptosis pathway, as measured by Fas, Fas ligand cellular expression, neither to increased expression of anti-apoptotic molecules of the intrinsic pathway such as bcl-2.

Alveolar macrophages are an important source of cytokines which participate in fibrogenesis. Little is known about the apoptotic profile of alveolar macrophages in IPF [4]. Intratracheal administration of apoptotic macrophages causes increased macrophage infiltration and apoptosis [13, 14] and in experimental models bleomycin induces alveolar macrophage apoptosis [15–17].

In lung biopsies of patients with IPF, fas was significantly expressed in macrophages compared to control [18]. In bleomycin-induced fibrosis increased macrophage expression of bcl-2 and bax proteins as well as caspases-1 and 3 has been described [19, 20]. In the present study, we did not find increased expression of bcl-2 or bax. This difference probably represents the discordance between bleomycin-induced fibrosis in animal models and pulmonary fibrosis in humans. Our present results are in accordance with previous observations of our group [21]. Moreover, immunochemistry depicts only a snapshot of a continuous, self-destructing process, without being able to describe the whole “pathogenesis story.”

Cytokines can influence macrophage apoptotic death: macrophage apoptosis can be increased by interferon-gamma and reduced by IL-4, IL-10 and TGF-b [22]. Mice deficient for macrophage-colony stimulating factor (M-CSF) develop less pulmonary fibrosis and have a decreased number of macrophages in their lungs after bleomycin installation. M-CSF was significantly of higher levels in the bronchoalveolar lavage of patients with IPF [23].

Since macrophages participate so actively in cytokine production and the development of inflammation and fibrosis, increased removal of macrophages from the fibrosis sites might be beneficial, especially if this procedure could be realized by a noninflammatory process such as apoptosis. Recent studies support such a hypothesis, showing that alveolar macrophage depletion is associated with reduction of fibrosis in animal models [24]. We assume that the decreased apoptotic rate of macrophages shown in the present study could be involved in the preservation of pulmonary fibrosis.

Macrophages are the professional phagocytes of apoptotic cells which can engulf apoptotic remnants [25]. Macrophages ingesting apoptotic cells release the anti-inflammatory cytokines IL-10 and TGF-b1 [26, 27]. Furthermore, apoptotic cells can produce IL-10 and TGF-b1 as well, thus enhancing the phagocytic capacity of macrophages [28–30]. Macrophages which phagocytose apoptotic bodies can also release proapoptotic factors that induce apoptosis of adjacent cells [31].

Bronchoalveolar lavage is a useful research tool for the study of interstitial lung diseases [6]. An increase of neutrophils and eosinophils in the bronchoalveolar lavage of patients with IPF correlates to worse prognosis [32–34]. Such a difference was observed between IPF patients and the control group in our study.

Pharmacological therapies have not been shown to offer a survival benefit in patients with IPF [35–37]. We have also reported that interferon-g and azathioprine plus corticosteroids have proven equally nonefficacious in preventing deterioration in IPF in a six month period [38].

We tried to correlate apoptotic markers' expression with parameters of respiratory function, as an attempt to identify a potential biomarker of the disease. Such a correlation could not be established except for the relation of the antiapoptotic factor bcl-2 signals with diffusing capacity for carbon monoxide after treatment. This is the first report to our knowledge of such a correlation. However, this correlation should be regarded in caution and reassessed in larger studies as its physiological rationale is unclear. At present, better biomarkers for IPF do exist and they include KL-6, surfactant proteins A and D. Soluble Fas in bronchoalveolar lavage and in serum has also been related to IPF severity [39].

Important limitations apply to our study: first, the number of patients included is relatively small. However, IPF is a rare disease and bronchoscopy is not performed in all cases [7].

Second, computerized image analysis is mainly applied in biopsies and there is limited data regarding its application in bronchoalveolar lavage. However, we preferred its use as it represents a more objective evaluation method compared to a semiquantitative measurement applied by the pathologist [40, 41]. In order to accumulate more data, not only did we measure staining intensity (which is more commonly measured in computerized image analysis) but also the total number of signals (objects) per optical field which represent positively stained cells.

Third, we did not examine the expression of other apoptotic factors such as TNF-a and TNF-a receptor or bak or bim of the extrinsic and intrinsic apoptotic pathway which might influence macrophage apoptosis [2]. We examined the expression of proteins that we thought would be more likely according to the literature, to be upregulated or downregulated in apoptosis of macrophages.

In conclusion, the present study showed that alveolar macrophages of patients with IPF exhibit a decreased apoptotic rate compared to normal subjects. We assume that macrophage resistance to apoptosis could be correlated to a continuous pathologic healing process observed in IPF.

## Figures and Tables

**Table 1:** Patients' baseline characteristics.

	Minimum	Maximum	Mean	Std. deviation
Age	54,00	80,00	69,05	6,20
Symptoms' duration (months)	2,00	48,00	14,15	11,65
FVC%	37,00	99,00	71,1	19,1
DLCO%	6,00	122,00	55,2	27,9
PaO_2_	58,00	95,00	72,09	11,54

**Table 2:** Bronchoalveolar lavage fluid parameters of IPF patients and control group at entry.

Cell type	IPF group (%) *n* = 20	Control group (%) *n* = 10	*P* value^∗∗^
Neutrophils	24,6 ± 4,6	6,1 ± 3,8	**0.001**
Macrophages	55,9 ± 6,9	81,4 ± 5,4	**0.001**
Lymphocytes	5,7 ± 3,7	12,6 ± 2,8	**0.001**
Eosinophils	10,8 ± 3,7	0,4 ± 1,0	**0.001**
Mast cells	2,6 ± 2,1	0 ± 0	**0.001**

Total cell count (×10^6^)	20,06 ± 3,03	26,3 ± 4,9	**0.001**

*P* < 0.05: statistically significant.

**Table 3:** Apoptotic markers' expression (CIA mean values) in patients before treatment and in control subjects.

	Control group [CI]	IPF group [CI]	*P*
ApoptAg density	100,1 [84,8–118,2]	124,8 [109,6–142,0]	**0.030**
ApoptAg area	580,03 [281,5–1193,7]	84,88 [40,8–175,2]	**<0.001**
ApoptAg (+) cells	78,8 [49,9–124,2]	11,1 [5,19–22,7]	**<0.001**
Bcl-2 density	149,8 [131,3–170,8]	151,7 [138,0–166,9]	0.883
Bcl-2 (+) cells	48,2 [26,1–88,5]	21,3 [12,9–34,5]	**0.030**
Bax density	134,6 [121,7–148,9]	131,0 [117,9–145,6]	0.771
Bax (+) cells	65,4 [37,2–114,5]	72,3 [53,9–96,7]	0.711
Fas ligand density	139,2 [129,2–150,1]	133,3 [126,1–140,9]	0.312
Fas ligand (+) cells	49,3 [30,1–80,5]	71,0 [51,7–97,3]	0.172
Fas density	163,5 [149,1–179,2]	151,3 [135,5–169,0]	0.291
Fas (+) cells	23,1 [13,5–39,1]	29,8 [18,9–46,7]	0.461

*P* < 0.05: statistically significant.

**Table 4:** Pulmonary function parameters before and after treatment with IFN*γ*-1b or AZA.

	IFN*γ*-1b group			AZA group		
	Before treatment	After treatment	*P*	Before treatment	After treatment	*P*
FVC (%pred)	73.8 ± 18.6	64.0 ± 23.8	0.096	68.4 ± 21.6	68.5 ± 19.6	0.977
FEV_1_ (%pred)	80.8 ± 19.4	70.7 ± 24.2	0.169	73.1 ± 19.5	72.1 ± 14.0	0.807
DLCO (%pred)	50.9 ± 39.4	37.5 ± 18.6	0.110	59.5 ± 15.7	61.8 ± 26.0	0.608
PO_2_ at rest	76.09 ± 9,60	72.66 ± 12.97	0.490	68,10 ± 12,48	64.90 ± 8.68	0.360

*P* < 0.05: statistically significant.

**Table 5:** Correlation of apoptotic markers' expression with respiratory function parameters before and after treatment in patients with IPF.

Before treatment		FVC	FVC%	FEV1	FEV1%	FEV1/FVC	DLCO	DLCO%	aFVC	aFVC%	aFEV1	aFEV1%	aFEV1/FVC	aDLCO	aDLCO%
ApoptAg (+) cells	Pearson	,010	−,215	,021	−,190	−,104	,046	,066	−,060	−,212	−,027	−,192	,139	−,219	−,234
	*P*	,966	,377	,931	,436	,672	,875	,821	,812	,398	,916	,446	,583	,473	,441
Apopt Ag area	Pearson	,021	−,206	,046	−,247	,134	−,377	−,340	−,069	−,236	−,002	−,242	,259	−,300	−,439
	*P*	,942	,460	,871	,374	,634	,283	,337	,806	,397	,994	,384	,351	,343	,153
ApoptAg density	Pearson	−,052	,177	,019	,240	,427	,042	,188	−,243	−,187	−,238	−,330	,348	−,517	−,538
	*P*	,854	,529	,947	,390	,113	,908	,602	,383	,504	,393	,230	,204	,086	,071
Bcl-2 (+) cells	Pearson	,388	,168	,365	,082	−,279	,204	,089	,449	,334	,462	,281	−,387	**,557**	,451
	*P*	,091	,479	,113	,730	,233	,466	,751	,054	,162	,046	,245	,102	**,039 **	,106
Bcl-2 density	Pearson	−,196	−,082	−,215	−,071	,144	−,086	-,015	−,058	,075	−,048	,105	,188	−,439	−,421
	*P*	,467	,762	,425	,794	,594	,780	,961	,838	,792	,866	,709	,502	,204	,226
Bax (+) cells	Pearson	,039	−,199	−,086	−,314	−,643	,314	,265	,105	−,021	−,015	−,125	−,330	−,066	−,024
	*P*	,872	,401	,718	,178	,002	,255	,340	,667	,932	,951	,611	,168	,823	,936
Bax density	Pearson	−,072	−,257	−,125	−,379	,269	−,329	−,247	,183	,109	,198	,060	−,078	−,435	−,368
	*P*	,790	,337	,646	,148	,314	,250	,395	,513	,699	,479	,832	,781	,182	,266
Fas (+) cells	Pearson	,040	−,180	,070	−,217	−,268	,234	,241	,037	,000	,103	,044	−,011	,119	,157
	*P*	,866	,448	,769	,359	,254	,402	,386	,879	,999	,674	,857	,965	,686	,591
Fas density	Pearson	−,303	−,109	−,324	−,018	−,025	,258	,317	−,243	−,047	−,327	,009	,001	,124	,229
	*P*	,254	,688	,221	,947	,926	,395	,291	,383	,867	,234	,974	,997	,732	,524
Fasl (+) cells	Pearson	−,153	−,369	−,176	−,310	−,385	,073	,050	−,230	−,357	−,283	−,309	,089	−,081	−,028
	*P*	,520	,110	,459	,184	,094	,796	,861	,343	,133	,240	,198	,718	,782	,924
Fasl density	Pearson	,195	,172	,223	,195	−,077	,215	,152	,102	,053	,072	−,021	−,171	,548	,431
	*P*	,486	,541	,424	,487	,784	,501	,637	,729	,858	,807	,942	,558	,127	,247

*P* < 0.05: statistically significant.

FVC: forced vital capacity, FEV1: forced expiratory volume in one second, DLCO: diffusing capacity for carbon monoxide, a: after six months of treatment.
